# A Systematic Review of Using Deep Learning Technology in the Steady-State Visually Evoked Potential-Based Brain-Computer Interface Applications: Current Trends and Future Trust Methodology

**DOI:** 10.1155/2023/7741735

**Published:** 2023-04-30

**Authors:** A. S. Albahri, Z. T. Al-qaysi, Laith Alzubaidi, Alhamzah Alnoor, O. S. Albahri, A. H. Alamoodi, Anizah Abu Bakar

**Affiliations:** ^1^Iraqi Commission for Computers and Informatics (ICCI), Baghdad, Iraq; ^2^Department of Computer Science, Computer Science and Mathematics College, Tikrit University, Tikrit, Iraq; ^3^School of Mechanical, Medical, and Process Engineering, Queensland University of Technology, Brisbane, QLD 4000, Australia; ^4^ARC Industrial Transformation Training Centre—Joint Biomechanics, Queensland University of Technology, Brisbane, QLD 4000, Australia; ^5^Southern Technical University, Basrah, Iraq; ^6^Computer Techniques Engineering Department, Mazaya University College, Nasiriyah, Iraq; ^7^Department of Computer Science and Information Technology, La Trobe University, Melbourne, VIC, Australia; ^8^Faculty of Computing and Meta-Technology (FKMT), Universiti Pendidikan Sultan Idris (UPSI), Perak, Malaysia; ^9^School of Computer Science, Universiti Sains Malaysia, Malaysia

## Abstract

The significance of deep learning techniques in relation to steady-state visually evoked potential- (SSVEP-) based brain-computer interface (BCI) applications is assessed through a systematic review. Three reliable databases, PubMed, ScienceDirect, and IEEE, were considered to gather relevant scientific and theoretical articles. Initially, 125 papers were found between 2010 and 2021 related to this integrated research field. After the filtering process, only 30 articles were identified and classified into five categories based on their type of deep learning methods. The first category, convolutional neural network (CNN), accounts for 70% (*n* = 21/30). The second category, recurrent neural network (RNN), accounts for 10% (*n* = 3/30). The third and fourth categories, deep neural network (DNN) and long short-term memory (LSTM), account for 6% (*n* = 30). The fifth category, restricted Boltzmann machine (RBM), accounts for 3% (*n* = 1/30). The literature's findings in terms of the main aspects identified in existing applications of deep learning pattern recognition techniques in SSVEP-based BCI, such as feature extraction, classification, activation functions, validation methods, and achieved classification accuracies, are examined. A comprehensive mapping analysis was also conducted, which identified six categories. Current challenges of ensuring trustworthy deep learning in SSVEP-based BCI applications were discussed, and recommendations were provided to researchers and developers. The study critically reviews the current unsolved issues of SSVEP-based BCI applications in terms of development challenges based on deep learning techniques and selection challenges based on multicriteria decision-making (MCDM). A trust proposal solution is presented with three methodology phases for evaluating and benchmarking SSVEP-based BCI applications using fuzzy decision-making techniques. Valuable insights and recommendations for researchers and developers in the SSVEP-based BCI and deep learning are provided.

## 1. Introduction

Artificial intelligence (AI) technology advancements, particularly in the brain-computer interface (BCI), are rapidly growing in medical and nonmedical applications. Patients who have been paralyzed after a stroke and those undergoing inpatient rehabilitation are aided by BCI technology in the medical field. The nonmedical applications are used in the internment applications such as video games [1, 2]. Brain-machine interfaces (BMIs) use electric signals from the brain to control electronic devices without using limbs. Those with complex neuromuscular problems, such as amyotrophic lateral sclerosis (ALS), have their brain impulses converted into actions by BCI technology to restore certain functions and abilities [2]. In BCI technology, various electroencephalographic (EEG) signals are used for steady-state visually evoked potential (SSVEP), movement-related cortical potential, motor imagery, and P-300 [3]. This field of research interests researchers in rehabilitation medicine and neuroscience, which utilize AI theory to study it [4].

SSVEPs produce natural responses to visual stimulation at specific frequencies. They are helpful in neurology and neuroscience research due to their excellence in signal-to-noise ratio and relative immunity to artefacts [2]. The SSVEP is a persistent response that the visual cortex produces after repeating retinal inputs with a specific frequency. Currently, SSVEP-based BCI applications are widely applied in the academic literature [5]. SSVEP implementation for BCI use cases is aimed at directly detecting brain activity and communicating messages to the outside world. SSVEP is a visually evoked potential (VEP) type that can be extracted from the attention of the subject, which focuses on a repetitive visual stimulus. Signal processing and other techniques like pattern recognition can determine the frequency and harmonics of SSVEPs [3]. The visual stimulus can be an LED or a pattern reversal stimulus, such as a checkerboard. Neural signals should be exploited by cognitive BMIs in more diverse areas that range from particular areas, both parietal and frontal, to complex prefrontal networks. The SSVEP-extracted feature may be constructed into the appropriate BCI applications more effectively using the deep learning technique. Accordingly, this research focuses on demonstrating and strengthening the contribution of deep learning methods in SSVEP-based BCI applications [6].

Natural responses to visual stimulation at specific frequencies are produced by SSVEPs [7]. Due to their excellence in signal-to-noise ratio and relative immunity to artefacts, they are helpful in neurology and neuroscience research. A persistent response, the SSVEP, is produced by the visual cortex after repeating retinal inputs with a specific frequency [3]. SSVEP-based BCI applications are widely applied in the academic literature. SSVEP implementation for BCI use cases is aimed at directly detecting brain activity and communicating messages to the outside world [8]. A visually evoked potential (VEP) type, the SSVEP, can be extracted from the attention of the subject, which focuses on a repetitive visual stimulus [9]. The frequency and harmonics of SSVEPs can be determined by signal processing and other techniques like pattern recognition [6]. The visual stimulus can be an LED or a pattern reversal stimulus, such as a checkerboard [9, 10]. Cognitive BMIs should exploit neural signals in more diverse areas that range from particular areas, both parietal and frontal, to complex prefrontal networks. The SSVEP-extracted feature can be constructed into the appropriate BCI applications more effectively using the deep learning technique [6, 11–13]. This research demonstrates and strengthens deep learning methods' contribution to SSVEP-based BCI applications.

To understand the issue and current research in this domain and to provide a clear understanding of the problems encountered, their solutions, and the main idea being pursued in this research, this introduction is structured in a question-and-answer format, starting with the following question:

Q1: “How does BCI base on SSVEP work?”

**Figure 1:**
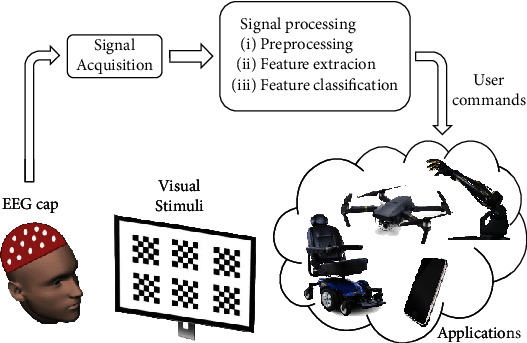
Functional model of an SSVEP-based BCI [19].

SSVEPs based on BCIs have gained popularity due to their high information transfer rate, ease of use, and minimal training requirements [14]. These responses are triggered by the stimulation of the retina at a specific frequency and can be detected through EEG on the visual cortex. Various fields have applied SSVEPs, including medicine, industry, communication, home automation, gaming, and robot and vehicle control [15]. To generate an SSVEP-based image, a target image is divided into smaller overlapping subimages presented as visual stimuli for a set period, flickering at a constant frequency [16]. Simultaneously, raw EEG data is collected from a wearable EEG device, and the EEG data corresponding to each subimage is extracted using signal processing techniques to represent a pixel of information [17, 18] (see Figure 1).

SSVEPs are found to be periodic responses to the rapid repetition of visual stimuli at frequencies between 1 and 100 Hz and are composed of harmonic frequencies [5]. Despite being utilized in BCI systems, long-term use can result in fatigue, limiting their potential applications [20]. Studies have been conducted to explore the potential applications of SSVEPs in cognitive and clinical neurosciences using deep learning techniques, including in areas such as visual attention, binocular rivalry, working memory, and EEG waves, as well as in the diagnosis of neurodegenerative disorders, schizophrenia, ophthalmic conditions, depression, autism, anxiety, stress, and epilepsy [18, 21]. This leads to the following question:

Q2: “How does deep learning contribute to SSVEP-based BCI applications?”

**Figure 2:**
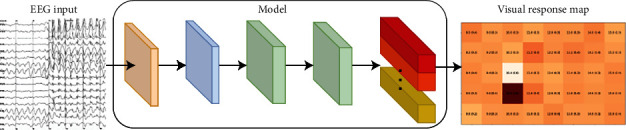
SSVEP-based deep learning model [27].

Deep learning techniques have recently made significant progress in pattern recognition and signal processing, particularly in computer vision and natural language processing [22, 23]. These techniques have also been applied to time series classification, including in the field of BCIs. The advantages of using deep learning in BCI research include processing raw brain signals directly, eliminating the need for time-consuming preprocessing and feature engineering, and capturing high-level features and latent dependencies through deep neural network structures [24]. As EEG signals are high-dimensional, deep learning techniques can extract more comprehensive EEG features than manually designed features [5]. The use of deep learning in BCI research has also led to algorithmic advances, such as data augmentation and transfer learning, which can reduce or eliminate the need for data acquisition and calibration. These advancements can significantly impact BCI research by allowing for the learning of complex patterns inherent in EEG and addressing the issue of insufficient data in certain datasets [25]. In recent years, deep learning techniques, particularly neural network-based methods such as convolutional neural networks (CNNs), have been successfully applied to various fields and utilized in the analysis of multichannel EEG signals for tasks such as the classification of visually evoked potential signals [20]. Unlike traditional methods, CNNs can perform automatic feature extraction and classification as an end-to-end decoding process. Several studies have demonstrated the effectiveness of using CNNs to improve the classification of scalp-EEG signals by combining EEGs from different channels and at different times through nonlinear operations on signals [26]. In the context of SSVEP, CNNs are structured with five layers: an input layer, a convolutional layer, a linear unit layer, a pooling layer, and a fully connected layer [14, 15]. These layers are organized into three dimensions: height, width, and depth [27] (see Figure 2).

Deep learning techniques are utilized in the integrated framework of SSVEP-based BCI applications to provide a self-learning procedure for regression and classification tasks, including speech, image, and video [28]. The pattern of producing high-level abstract aspects utilizing low-level data integration and recognizing properties specific to the data arrangement is a significant characteristic of deep learning [29]. Normal shallow learning techniques, such as radial basis function (RBF) or BP networks, are overcome in their inability to present features by deep learning [30]. Complex layers, including hidden ones, are more involved in top-down SSVEP-based cognitive BCIs than those with physically driven bottom-up ones, with even the physiological processes of such layers being unknown [13, 31–36]. This issue can be solved by implementing deep learning, which provides more optimal decoding performance by learning from raw and image data to decode SSVEP brain signals through understanding each SSVEP stimulus feature [10, 36, 37]. The critical analyses of current academic literature reviews for deep learning in SSVEP-based BCI applications are discussed further as it is significant to various neurology and neuroscience research areas. In order to capture the current state in this multidisciplinary field, this study clarifies and discusses the latest published reviews from the technical and scientific perspectives of deep learning and SSVEP-based BCIs. Therefore, the third question is as follows:

Q3: “What is the current literature review of SSVEP-based BCI applications?”

A review of training environments with motor imagery BCI is discussed by researchers [13]. Additionally, studies [13, 33] review the background of existing studies related to wheelchair control based on BCI for disability and map the literature study into an intelligible taxonomy. The development of motor imagery EEG based on classification BCI systems is also studied by researchers [32, 33]. While the existing studies cover several important aspects, integrating deep learning and SSVEP-based BCI applications are overlooked. To the best of the authors' knowledge, neither a review study nor a systematic review study discussing the utilization of deep learning methods in SSVEP-based BCI applications has been presented [3]. This research is aimed to filling this gap by examining different perspectives of deep learning and SSVEP-based BCI applications. The critical research facets of this domain are evaluated by providing researchers with an understanding of current deep learning trends and their applications in deploying SSVEP-based BCI applications. In-depth analyses were conducted to determine the most effective methods for collecting and storing data for this study, including a review of pattern recognition techniques applied to various research requirements [13]. The challenges and requirements for making SSVEP-based BCI applications trustworthy as current trend research are discussed, and recommendations for researchers and developers are presented in five important directions. Methods and solutions to assist SSVEP-based BCI researchers in improving available deep learning methods and techniques, overcoming issues, and increasing the usability of SSVEP-based BCI deployment for people with and without impairments are offered. Finally, a proposed solution for selecting the optimal SSVEP-based BCI application using a new integration methodology phase is presented. The contributions of this systematic review are as follows:

1. A thorough understanding of current deep learning trends and how they are applied to SSVEP-based BCI applications is provided for researchers
2. A review of pattern recognition techniques applied to various research requirements is presented
3. The challenges and requirements for making SSVEP-based BCI applications trustworthy as current trend research are discussed
4. Recommendations for researchers and developers are offered for improving the available deep learning methods and techniques, overcoming issues, and increasing the usability of SSVEP-based BCI deployment for people with and without impairments
5. A proposed solution for selecting the optimal SSVEP-based BCI application is presented using a new integration methodology phase

## 2. Method

**Figure 3:**
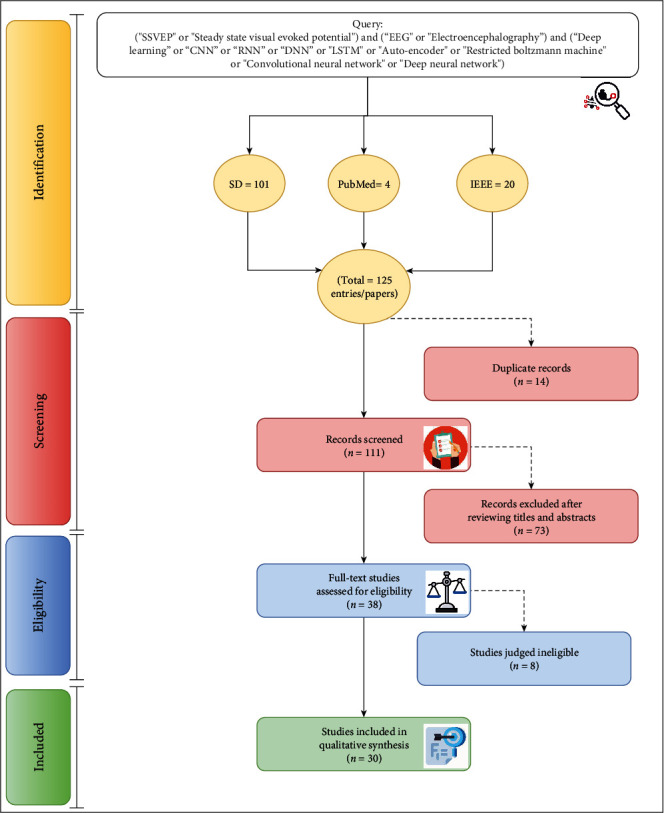
Schematic of the approach to identify, screen, and include relevant studies.

This study used the Preferred Reporting Items for Reviews and Meta-Analyses (PRISMA) to conduct the investigation, as shown in Figure 3 [31, 38, 39]. The researchers avoided relying on a single database to find literature in the review article, as all relevant references may not be included, so a supplementary search is often necessary [32, 40]. According to studies [36, 41–43], a review should be conducted across multiple databases to capture the majority of publications. The study used the ScienceDirect, PubMed, and IEEE databases for a detailed literature review of articles between 2010 and 2021. These databases extensively publish correlated articles and address conceptual, scientific, and clinical aspects from multidisciplinary viewpoints [44–46]. The authors formulated a Boolean search approach based on keywords associated with “steady-state visual evoked potential” (such as steady-state visual induced potential OR SSVEP) and those associated with electroencephalography (such as electroencephalography or the abbreviation EEG) and keywords with deep learning (such as RNN, LSTM, DNN, deep neural network, convolutional neural network, deep learning, CNN, and restricted Boltzmann machine, among others). The criteria for selecting relevant articles are as follows:

1. The article is written in English language
2. The article is a journal or conference paper
3. The study with SSVEP is based on an EEG device for data collection
4. The BCI modality is based on SSVEP
5. The AI algorithm employed to identify the SSVEP patterns is based on deep learning techniques

Not included articles are research works that focus on non-EEG devices, non-SSVEP brain signals, and nondeep learning-based applications. Three researchers followed the selection criteria to identify abstracts and titles, ignoring duplicates. They then evaluated the complete text of the likely relevant works. Three other researchers completed a process review comprising an assessment of the data in the screened papers. The authors were consulted, and the authenticity and relevance of the papers were assessed and validated. The research also implemented two screening techniques to identify articles relevant to the SSVEP domain using a deep learning approach for several BCI applications. The first screening process filters articles based on the title and abstract text [47–50]. The other filter comprises an extensive assessment of the complete articles. The data gathering process proposed by existing researchers is thoroughly assessed to review, providing insight and perspective from several significant researchers. This approach is aimed at enhancing the consistency and robustness of the study [51].

## 3. Systematic Results and Discussion

**Figure 4:**
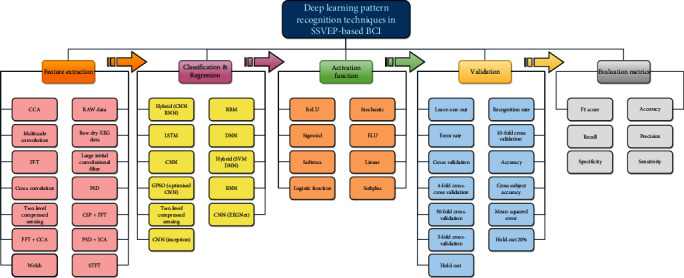
The existing applications of deep learning pattern recognition techniques in SSVEP-based BCI.

This section presents and describes the existing deep learning techniques in the literature for SSVEP-based BCI applications. An overview of existing feature extractions, classifications, activation functions, validation methods, and the achieved classification accuracy with detailed information regarding each subprocess is provided in Figure 4. In this literature, the CCA method for feature extraction is used in twelve existing works. Among them, the CNN technique is used by six researchers, as convolutional correlation analysis is utilized to enhance the performance of SSVEP-based BCI [15]. In contrast, CNN decodes the brain signal in BCI speller applications [52]. The frontal and occipital EEG features are fused to detect a “brain switch” using a CNN by the study in [53]. Asynchronous SSVEP signals are classified using compact CNNs [54]. The modular continuous restricted Boltzmann machine is used for SSVEP-based BCI applications [55]. Additionally, CNN is used to enhance the detection of SSVEP in the presence of competing stimuli [56]. On the other hand, the CCA with hybrid (CNN and RNN) technique for signal classification is used in only two studies [10, 28], in which the former is used for the detection of asynchronous steady-state motion visual-evoked potential and the latter for classifying the SSVEP brain signal in the time domain. Additionally, the CCA is used with LSTM in [45] for classifying multiflicker-SSVEP in single-channel dry-EEG for low-power/high-accuracy quadcopter-BMI systems. In the study [27], the CCA with the RRN classifier is used in an SSVEP-based BCI system for user authentication in a personal device. The CCA is also used with EEGNet and ensemble learning to improve the cross-session classification of SSVEP-based BCI from Ear-EEG. In research [6], singular spectrum analysis (SSA) is used to separate random and periodic EEG components, and the SSA parameters are optimized using the skewness coefficient and Spearman correlation. Advanced signal processing techniques, such as CCA and SSA, can improve the accuracy and robustness of BCI systems. Additionally, using machine learning algorithms, such as RRN and MLR, can further enhance the performance of these systems. A noninvasive BCI based on a CNN has been proposed in [57] for virtual environment (VE) navigation. SSVEP properties in EEG data are distinguished in real time by CNN to control the navigation interface. The proposed approach has been examined by walking in an immersive and believable virtual environment (VE), which enhances the participant's involvement and perception of the VE. In [58], the feasibility of using a CNN to interpret human EEG responses for authentication purposes is investigated. Specifically, biometrics are composed of low-frequency components of the SSVEP, which include stable and personalized patterns. The evaluation of the study [58] utilizes the distinguishing capacities over several parameter combinations to optimize the CNN model. Additionally, the authors analyze how the duration of EEG data affects authentication performance. Frequency domain methods, such as the fast Fourier transform (FFT), have been widely adopted in BCI modalities for extracting signal features from brain signals. Many studies have utilized this process in their feature extraction procedures in the current literature. For example, in [37], deep learning is applied to a top-down control strategy utilizing SSVEP brain signals. At the same time, [59] employs a convolutional neural network (CNN) for classifying SSVEP in an ambulatory environment. Additionally, [60] utilizes deep separable CNNs in the SSVEP framework. Furthermore, studies such as [59, 61] and [10, 62] utilize the FFT in conjunction with CNNs in BCI speller applications. However, in virtual environment (VE) applications, such as [54], the FFT is used in conjunction with CNNs for extracting brain signal features while controlling walking in the VE. Additionally, [20] proposes the multiharmonic linkage CNN (MHLCNN) model for SSVEP and SSMVEP signal classifications. Furthermore, studies such as [10, 15, 26, 28, 53, 56, 63, 64] utilize CNNs for person identification and improving the performance of SSVEP-based BCI systems, respectively. Researchers have employed various feature extraction methods in conjunction with their deep learning models for extracting SSVEP brain signal features. For example, cross-correlation [65] has been used for person authentication using SSVEP visual stimulation, while Welch's method [66] controls mobile robots in a brain-based teleoperation system. Additionally, two-level compressed sensing [30] has been employed in SSVEP-based BCI systems, and CNNs alone have been used in real-time humanoid robot navigation using SSVEP stimuli [67]. However, other studies have used raw EEG data to extract SSVEP signal features for a CNN-based user authentication system [61] and classify SSVEP-based CNN applications [68]. In the time-frequency domain, short-time Fourier transform (STFT) with CNNs has been used to classify SSVEP responses in a BCI framework [17, 69]. Multiscale convolution [19] has also been utilized to extract SSVEP features according to multiple frequencies in SSVEP stimulations. In terms of hybrid feature extraction methods, which are also widely used in BCI applications, studies such as [70] have employed a combination of power spectral density (PSD) and independent component analysis (ICA) for feature extraction and feature reduction to minimize the dimensionality of the BCI signal. Furthermore, in [14], FFT and CCA have been used to extract BCI system SSVEP signal features.

## 4. Comprehensive Mapping Analysis

In this study, various data are gathered using the literature and devised a classification based on the article bibliography, data acquisition method, and brain signal assessment. The first category comprises academicians' nationalities and publication years, followed by the word cloud. EEG instrument category, channel name and number employed for capturing brain signals, and the subject count are involved in data gathering in the second category. The third category contained the feature extraction technique for extracting the dominant features of the SSVEP brain signal, the deep learning approach used in distinguishing the brain signal, the classification accuracy system, and the advantages of the proposed system, framework, or algorithm. However, the review method suffers from issues of objectivity and reliability. Previous literature strongly recommended the adoption of bibliometric analysis to address the mentioned issues [8]. There are many ways to perform a bibliometric analysis, for example, RStudio and VOSviewer. This study adopted these techniques to reorganize the previous literature's findings, summarize the literature's findings, and provide insight to academics and practitioners about the previous works. Therefore, bibliometric analysis helps keep up with current and published research. The following subsections describe the results of the mapping analysis.

### 4.1. Word Cloud

**Figure 5:**
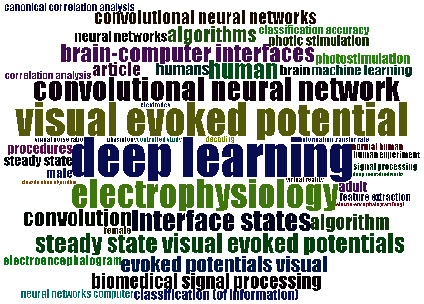
Word cloud.

The word cloud shows the most common and crucial terms from existing studies [71]. In order to synthesize the overview of those discoveries and rearrangement of information, Figure 5 provides key phrases in the findings of previous studies.

Figure 5 displays various keywords relevant to this field of study. The larger the keywords, the more frequently they appear in the literature. In contrast, the smaller the size of keywords, the less frequently they appear in the literature. In this context, SSVEP, BCI, deep learning, and EEG are several significant vital topics in the previous studies that we are looking into. Most of the studies have attempted to apply deep learning techniques to SSVEP-based BCI applications, especially in the usage of EEG. The study's findings indicate that feature extraction, classification, activation function, validation, and accuracy of the deep learning techniques are critical factors for the emergence of SSVEP-based BCI applications in medical use cases.

### 4.2. Cooccurrence

**Figure 6:**
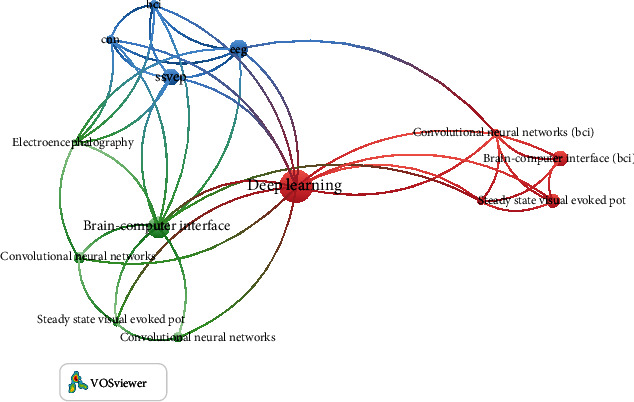
Cooccurrence.

Cooccurrence networks refer to common words established by existing studies [72]. Cooccurrence analysis is a semantic network that provides critical clues to researchers about the conceptual structure of a field of specialization.  Figure 6 shows cooccurrence networks and identifies common keywords.

The cooccurrence illustrates the topics' network consisting of lines and knots. Large knots represent the most common themes. Regarding SSVEP-based BCI applications in medical fields, feature extraction, classification, activation function, validation, and accuracy are integral to SSVEP, BCI, EEG, and deep learning. This cooccurrence provides a clear overview of the research area's related themes and conceptual structure. It also creates opportunities for researchers to identify the gaps in this research field. By capturing the common repetition of keywords for SSVEP-based BCI applications, researchers can apply data networks to facilitate attempts to reorganize the available information and results.

### 4.3. Trending Topics

**Figure 7:**
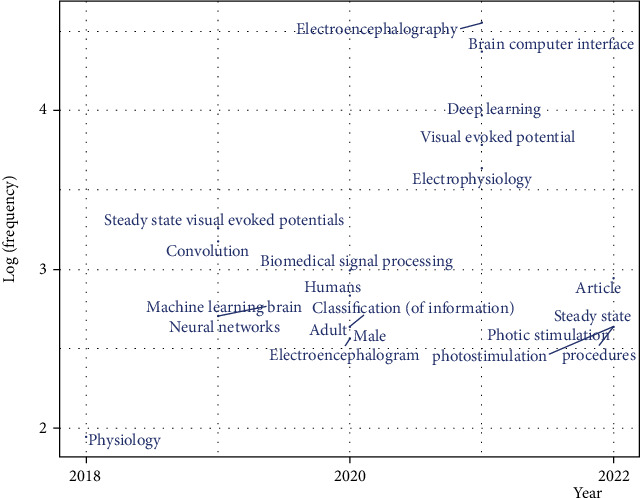
Trend topics in terms of the theoretical and practical aspects.

Trending topics are the most important topics dealing with a certain subject matter. Trending topics represent concepts and elements analyzed in the existing literature for deep learning techniques, SSVEP, BCI, and EGG.  Figure 7 shows the essential critical words adopted by existing studies about SSVEP-based BCI applications and the relationships between them.

Trending topics can support the research taxonomy because they illustrate and confirm that most studies related to SSVEP-based BCI applications focused on medical use cases. As shown in Figure 7, the most frequently used keyword is SSVEP, followed by BCI, as these two are closely related in this research field. The keywords indicate that the authors who investigated the SSVEP-based BCI applications were also interested in those areas. Trending topics can support the search ranking by clarifying the percentage of keywords.  Figure 7 shows the most relevant elements of the organized text data. This procedure prevented academics and recipients comparing the different components from finding the similarities and differences between the larger and bolder words. Furthermore, trending topics have provided huge opportunities for researchers to understand the importance of SSVEP-based BCI implementation in any field by grouping words of various sizes in terms of critical factors.

### 4.4. Factorial Analysis

**Figure 8:**
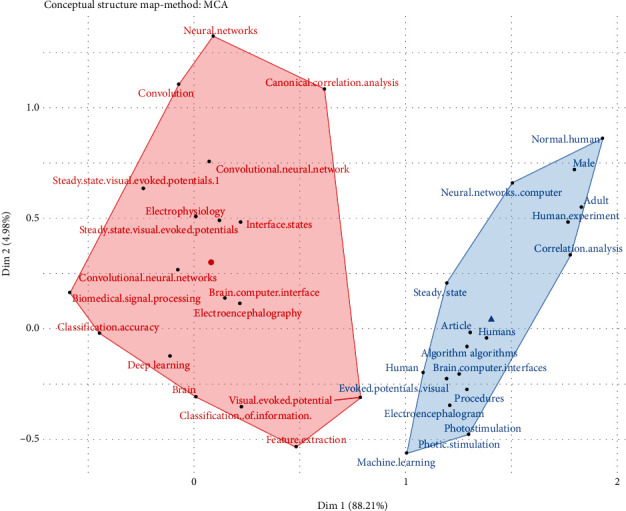
Factorial analysis in terms of theoretical and practical aspects.

The factorial analysis computes the similarity index, which allows the user to normalize bibliographic coupling, cooccurrence, and cocitation, a similarity measure. The factorial analysis was used to map the conceptual structure of a field based on the frequency of words in a particular bibliographical group. Such an analysis provides insight to the researchers into understanding the relationship between the main topic and emerging subfields. The size of nodes shows the frequency of occurrence of keywords. To this end, a factorial analysis has been used to understand the relationship between the topics which applied SSVEP-based BCI, as shown in Figure 8.

Two clusters have been shown based on distinct colors. Each cluster is a simultaneous keyword in the studies being sampled. The red cluster is the largest network of keywords comprising the main topic of SSVEP and BCI applications. The literature which has adopted significant deep learning techniques has been associated with other nodes such as feature extraction, classification, activation function, validation, and accuracy. The blue cluster provides insight into EGG and the accuracy of BCI applications in the medical field. Investigating factorial analysis provides the developing blocks for ongoing research and development in the SSVEP and BCI applications.

### 4.5. Collaboration Map

**Figure 9:**
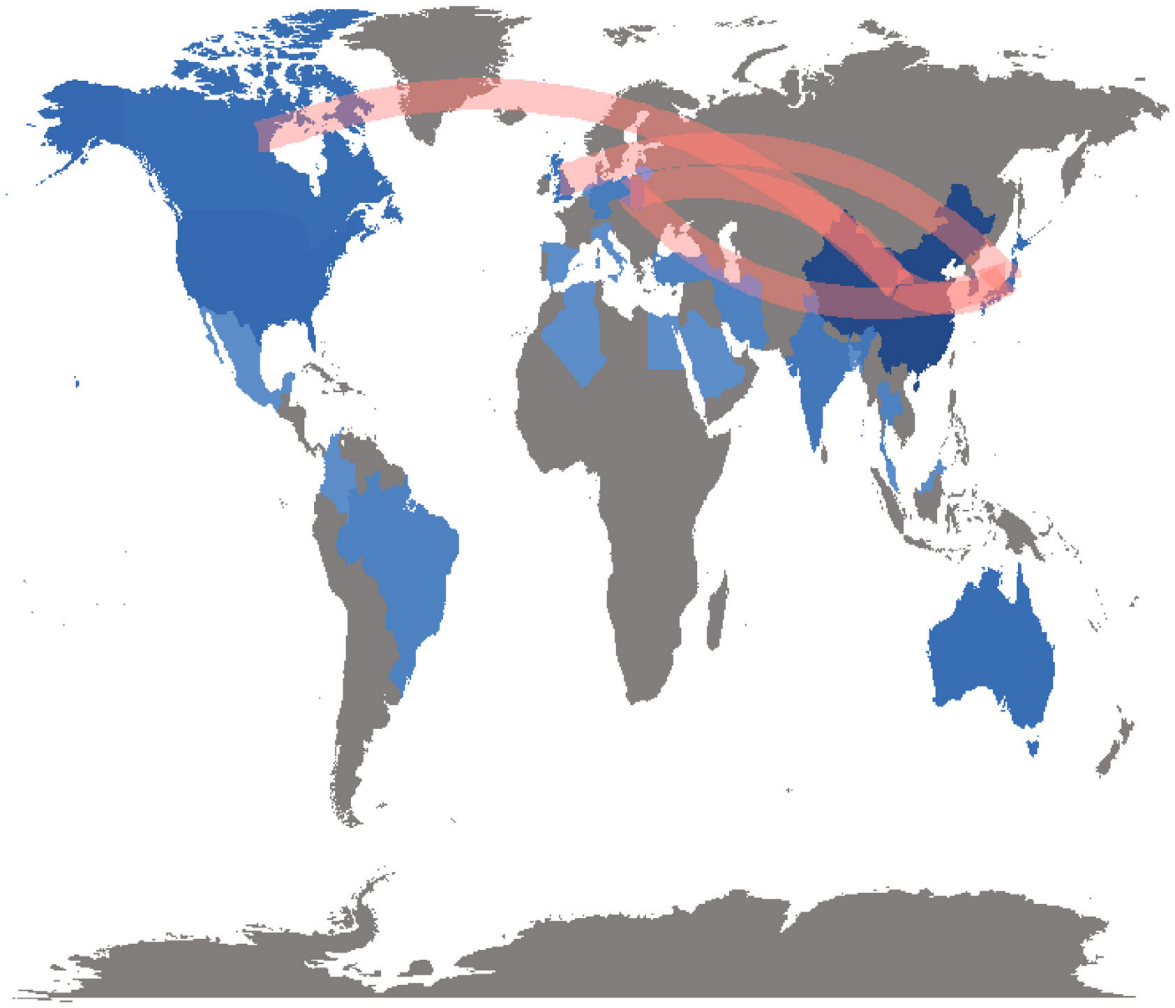
Collaboration world map.

The country collaboration map shows the scientific cooperation network between universities, countries, and authors. Coauthorship increases countries' and researchers' skills and experience in developing a specialization field [73].  Figure 9 presents a country collaboration map for SSVEP in healthcare.

The figure consists of three colors. Dark blue indicates the countries with the most scientific production, light blue represents little scientific production, and grey indicates the lack of scientific production. In addition, the red line represents scientific cooperation between countries.  Figure 9 confirms that scientific cooperation between the European and American continents enhances the SSVEP techniques in the healthcare sector. However, there is a considerable lack of scientific cooperation between the Asian, European, African, and American continents. Moreover, the lack of scientific cooperation between countries indicates a lack of experience and knowledge regarding the ethical issues in SSVEP applications. Therefore, policymakers and academics in the continents mentioned above should seek more innovative ways to stimulate and enhance scientific cooperation and benefit from the expertise and skills in this field in the European and American continents.

## 5. Current Challenges: Trustworthy Deep Learning in SSVEP-Based BCI Applications

Deep learning technology has become an important part of BCI applications that use SSVEPs [74]. These systems can greatly enhance our daily lives, but they also have the potential to cause harm to users or society, either directly or indirectly. Therefore, ensuring that these systems are safe, reliable, and trustworthy is essential [75, 76]. Trustworthy deep learning technology in these applications can be achieved through several measures, including thorough testing and evaluation, transparent design and decision-making processes, and robust security and privacy measures. By prioritizing these considerations, we can ensure that these systems are effective, responsible, and ethical in their use [77]. Accordingly, several requirements can help to ensure the trustworthiness of deep learning technology in BCI applications based on SSVEPs [78–80]. Some of these requirements include the following:

- Thorough testing and evaluation: deep learning systems should be tested and evaluated extensively to ensure that they are accurate, reliable, and effective. This can involve simulated and real-world testing to cover many scenarios
- Transparent design and decision-making processes: the design and decision-making processes behind these systems must be transparent so that users and other stakeholders can understand how the systems work and how decisions are made
- Robust security and privacy measures: these systems should have strong security measures to protect against unauthorized access or misuse. They should also have strong privacy protections to ensure that user data is handled responsibly and in compliance with relevant regulations
- Ethical considerations: deep learning systems should be designed and used ethically, considering the potential impacts on society and individual users. This can involve considering issues such as bias and diversity in the development and use of the systems
- User-centered design: these systems should be designed with the needs and preferences of the users in mind to ensure that they are easy to use and provide a positive user experience
- Explainability: it should be possible to understand and explain the decision-making processes of these systems so that users and other stakeholders can understand how they work and why certain decisions are made
- Responsiveness to change: deep learning systems should be able to adapt and learn over time to continue to be effective even in changing environments or as new data becomes available
- Responsiveness to feedback: these systems should be designed to be responsive to user feedback and adapt to changing user needs and preferences over time
- Scalability: deep learning systems should be able to handle large amounts of data and be able to operate effectively at scale

Several challenges can arise in developing and deploying trustworthy deep learning technology in BCI applications based on SSVEPs [81–83]. Some of these challenges include the following:

- Complexity: deep learning systems can be complex and may require specialized expertise to develop and maintain. This can make it challenging to ensure that these systems are reliable and trustworthy
- Bias and diversity: deep learning systems can be prone to bias if they are trained on biased data or are not designed to consider diverse perspectives. This can lead to unfair or discriminatory outcomes and undermine trust in these systems
- Security and privacy: ensuring the security and privacy of user data is a major challenge in developing deep learning systems, particularly in the context of BCIs, which may involve sensitive personal information
- Explainability: it can be difficult to understand and explain deep learning systems' decision-making processes, making it challenging to ensure that these systems are transparent and trustworthy
- Ethical considerations: deep learning systems can have unintended consequences and may have potential impacts on society and individual users. Ensuring that these systems are used ethically and responsibly can be a challenge
- Inability to adapt to change: if the system cannot adapt and learn over time, it may become less effective in changing environments or as new data becomes available
- Lack of scalability: if the system cannot handle large amounts of data or operate effectively at scale, it may not meet the needs of a growing user base or handle increasing amounts of data

## 6. Recommendations to Researchers and Developers

This study opens a way for future researchers to enhance this research field and cope with the existing studies. Future works can be focused on optimization in the pattern recognition models and the implementation of hybrid techniques to maximize the performance of the developed models. Additionally, the generalization issue can be addressed in future research to develop a generalized model that can fit any subject. These future works might help the researchers and the developers to deploy the deep learning-based BCI-SSVEP pattern recognition system in real-time systems. The future works can be directed into many paths and aspects, such as real-time implementations, optimizations, generalization, hybrid techniques, recognition of brain signals, and robotic rehabilitation system. These aspects are further elaborated in detail in the following subsections to portray future trends.

### 6.1. Real-Time Implementation

The real-time SSVEP categorization setup for exoskeleton and robotic cars must be addressed in future studies [28]. A real-time CNN implementation must be devised to regulate an exoskeleton, specifically the lower limb, and assess performance for healthy individuals and groups to determine the feasibility of gait rehabilitation. Nevertheless, a CNN classifier independent of subjects might provide a productive approach because it helps reduce training duration [59]. Assessing the influence of various window dimensions must be considered for future studies. Moreover, real-time setup and overlap concerning the remote operation of mobile robots should be assessed. A simple and highly accurate drone controller should be developed for a larger number of participants by using more complicated drone movements, its effectiveness should be verified, and the system should be implemented using lower-cost hardware that utilizes little power [63].

### 6.2. Optimization

Different deep learning techniques should be examined to enhance top-down SSVEP BMI's decoding accuracy [37]. Optimizing the hyperparameter is one of the important research directions. For example, CNN hyperparameters like network layer distribution, feature map size and count during convolution, and convolutional layer count are recommended evaluation areas. Further, several hyperparameters can be modified for performance improvement, including the learning rate, weight initialization methods, regularization-related methods, the number of iterations, and attenuation function. Optimizing parameters might provide a significantly positive effect on deep learning efficacy, leading to more significance on hyperparameter improvement specific to deep neural systems, which are already being explored extensively [70].

### 6.3. Generalization and Hybrid Technique

Enhancing generalization efficacy for more test subjects is a recommended future work direction. Setting complexity, teleoperation duration, and robot-environment interaction should be studied [67]. Further studies should involve large datasets for the classification and generalization across subjects in the different deep learning approaches [68]. Further research is needed to investigate how to adapt or use combined CCA directly to handle asynchronous operations better [54]. Other forms of the MCRBM framework should be assessed to enhance the performance of the SSVEP-BCI approach [55].

### 6.4. Robotic Rehabilitation Systems

An SSVEP-based BCI system with an integrated rehabilitation robot system has been proposed. The subject's movements were perceived as commands to the robot system to help the upper extremities engage in physical therapy-like training [67]. The existing study focused on determining whether performance in a BCI based on SSVEP response produced by visual attention was affected by attention to real-world activity. However, the asynchronous BCI system should be improved rather than the current synchronization SSVEP-based BCI. The study can be further directed into validating this integrated rehabilitation approach with a group of patients who have upper limb paralysis [66].

### 6.5. Recognition of Brain Signal

An SSVEP-BCI implementation has several IC states. High recognition accuracy is challenging because prevalent recognition approaches use the threshold mechanism and suffer from statistical issues like concurrent low false positives and high true positives [10]. Several obstacles still exist despite significant technological advancements. The BMI control signals are generated by the primary sensory-related processing of the brain areas, which are the most commonly used. However, utilizing these signals only limits the decodable human intentions' range. Thus, for better controlling multiclass BCIs, further brain activity sources need to be explored [37]. This can be enhanced in future works as ambulatory BCI regulation provides reduced performance levels compared to a static reference because of deterioration caused by subject movement, head movement, sound, walking speed, and electric influence of exoskeleton motors, leading to different user characteristics [59]. Deep learning schemes use handcrafted processes. Complete training for feature identification and extraction was not implemented. EEG data has high volatility, overloading convolutional feature identifiers. These aspects hinder system convergence [60]. These issues need a thorough study in the future by focusing on hardware inputs that can be reduced using single EEG electrodes. It is suggested to apply this approach due to its cost-efficiency and simplicity of the system [63]. Paste-free dry electrodes with lesser contact resistance concerning the electrode and scalp surface can help participants by reducing cleaning effort after the procedure [63]. Additional brain activity sources need to be explored. In BCIs, a new potential field could be opened by the brain activity associated with top-down cognitive functions [37].

## 7. Critical Review and Unsolved Issues of SSVEP-Based BCI Applications

The applications of SSVEP-based BCIs and their effects on individuals are discussed in this section of the research paper. The examined researches how SSVEP-based BCIs can be utilized to help individuals in various settings and explores the potential implications [84]. This section discusses and highlights two main challenges regarding the future trust methodology of evaluating and benchmarking SSVEP-based BCI applications.

### 7.1. Development Challenges Based on Deep Learning Techniques

There are several unsolved issues when using DL technology in SSVEP-based BCI applications. These include the following:

1. One of the major challenges in utilizing deep learning for SSVEP-based BCI applications is obtaining high classification accuracy [79]. SSVEP signals tend to be weak and can be easily overwhelmed by noise originating from various sources, such as eye movements, muscle activity, and electrical interference. This noise can obscure the SSVEP signals, making them difficult to detect, which leads to low classification accuracy. Several solutions have been proposed to address this issue, such as utilizing advanced signal processing techniques, applying spatial filtering to improve the signal-to-noise ratio, and using ensemble methods to combine the outputs of multiple classifiers. Additionally, research is being conducted to develop deep learning models that are more robust to noise and have greater generalization capabilities
2. The limited frequency range of SSVEP signals can restrict the number of commands a BCI system can detect [85]. SSVEP signals are created in response to visual stimuli that flicker at a specific frequency and typically have a frequency range between 5 and 25 Hz. This means that a BCI system using SSVEP signals can only detect commands associated with visual stimuli that flicker within this frequency range. This limitation reduces the number of commands detected by the BCI system and the number of stimuli that can be presented to the user. Additionally, the frequency range of SSVEP signals can vary among individuals, leading to variability in the system's performance across different users. To overcome this limitation, researchers have proposed methods to detect SSVEP signals at higher frequencies by using frequency-tagged visual stimuli and advanced signal processing techniques to extract SSVEP signals from EEG data
3. The high variability of SSVEP signals between individuals poses a significant challenge in creating a generalizable BCI system. Factors such as visual acuity, cognitive abilities, and brain anatomy can lead to significant differences in SSVEP signals among individuals, making it challenging to develop a system that works well for many users [86]. To address this issue, some researchers have suggested using personalized calibration methods, such as individualized stimuli or adapting the model to each user's specific characteristics. Additionally, advanced signal processing techniques, such as ICA or CSP, have been proposed to extract more robust features to intersubject variability. Still, these methods can be less effective and more complex to implement. More research is needed to overcome this limitation, understand the causes of intersubject variability, and develop strategies to reduce it
4. Head movements can greatly affect the performance and stability of SSVEP-based BCI systems as they can alter the quality of SSVEP signals. Head movements can cause changes in the EEG signals, such as shifts in the position of electrodes, changes in reference and ground electrodes, and changes in the eyes' position relative to the stimuli [87]. These changes can lead to a reduction in the SSVEP signals' amplitude and frequency, which makes them more difficult to detect. This can decrease the performance of the BCI system and make it less dependable. Several methods have been suggested to address this problem, such as using advanced signal processing techniques to correct for head movements' effects, using eye-tracking systems to monitor gaze position and compensate for changes in SSVEP signals, and using head-mounted displays to keep stimuli in a fixed position relative to the eyes. Additionally, some researchers are exploring using deep learning models to detect and correct head movements in real time
5. Using a limited number of EEG channels in SSVEP-based BCI systems can restrict the amount of information that can be obtained from the brain. Typically, SSVEP-based BCI systems use only a small number of EEG channels, ranging from 4 to 8, to detect SSVEP signals which can limit the system's spatial resolution, making it harder to differentiate between different SSVEP signals [88]. Furthermore, it also limits the ability to detect SSVEP signals from different brain regions, which can be important for broadening the range of applications of BCI systems. To address this limitation, researchers have suggested using advanced signal processing techniques such as ICA and CSP to extract features that are less affected by the limited number of channels. Additionally, some researchers have proposed using high-density EEG systems with up to 128 channels to enhance the system's spatial resolution and improve the detection of SSVEP signals
6. The lack of interpretability in deep learning models is a significant challenge when using them in SSVEP-based BCI systems [89]. These models are considered black boxes due to their complexity and nonlinearity, making it hard to understand how they make decisions and which features they use to classify SSVEP signals. This can make it difficult to debug the models and ensure they make decisions based on the correct features. It can also make it challenging to ensure that the models are not making decisions based on irrelevant or biased features. To overcome this limitation, researchers have proposed using interpretable machine learning methods such as decision trees and rule-based systems or methods to visualize and comprehend the internal representations of deep learning models like saliency maps, activation maximization, and layer-wise relevance propagation
7. Obtaining a sufficient amount of labeled EEG data is a key challenge when training and fine-tuning deep learning models for SSVEP-based BCI applications [17]. These models require a large amount of data, which can be difficult to acquire. Typically, collecting EEG data for BCI applications requires participants to perform a specific task, such as looking at a flickering stimulus, while the data is being recorded. This process can be costly and time-consuming, making it hard to ensure that the data is of high quality. Furthermore, collecting EEG data from a diverse population can be challenging, making it hard to develop models that generalize well to different users. To address this issue, researchers have suggested using transfer learning and domain adaptation techniques to fine-tune pretrained models using a smaller amount of labeled data. Additionally, researchers have proposed using synthetic data, such as computer-generated EEG signals, to increase the amount of labeled data available for training and fine-tuning models
8. Real-time performance is a significant issue when using deep learning models for SSVEP-based BCI systems, as these models can be computationally intensive [90]. Deep learning models typically have many parameters that require significant computational resources to train and evaluate. Furthermore, the inference stage of deep learning models can also be computationally expensive, as it requires many matrix multiplications and nonlinear operations. This can make it challenging to implement these models in real-time BCI systems, as the EEG data needs to be processed in real time to provide timely feedback to the user. To end this limitation, researchers have proposed using techniques such as model compression, quantization, and hardware acceleration to reduce the computational cost of deep learning models. Alternatively, researchers have proposed using lightweight models such as shallow neural networks or decision trees, which are more computationally efficient than deep learning models

In addition to the above issues, developing SSVEP-based BCI applications presents several challenges, particularly considering the criteria of security [28, 58, 64], robot control [52, 65–67], BCI framework [37, 56, 59], drone control [55, 66], and BCI speller [62]. More explanation for each criterion explains as follows:

- Security: SSVEP-based BCI systems are vulnerable to signal spoofing, in which an attacker generates an artificial signal to impersonate a genuine user. Additionally, SSVEP-based BCI systems also face the challenge of ensuring the privacy of the users' brain signals, which may contain sensitive information
- Robot control: SSVEP-based BCI systems are challenging to apply in real-world robot control scenarios because of the variability of the SSVEP signals across different users and environmental conditions. Additionally, achieving accurate and responsive control of robots using SSVEP-based BCI systems is challenging and may require significant computational resources
- BCI framework: developing a robust and reliable BCI framework for SSVEP-based systems is a challenging task. It requires integrating components such as signal acquisition, feature extraction, classification, and feedback, which may be affected by different factors such as noise, artefacts, and variability of the SSVEP signals
- Drone control: the control of drones using SSVEP-based BCI systems is challenging due to the high speed and dynamic nature of the drones. Additionally, achieving accurate and responsive control of drones using SSVEP-based BCI systems is challenging and may require significant computational resources and advanced signal processing techniques
- BCI speller: SSVEP-based BCI speller applications are challenging to develop because of the high variability of the SSVEP signals across different users and environmental conditions. Additionally, it is challenging to achieve accurate and responsive control of the speller using SSVEP-based BCI systems, which may require significant computational resources and advanced signal processing techniques

### 7.2. Selection Challenges Based on MCDM

This review has highlighted that the use of deep learning techniques in SSVEP-based BCI applications has increased in recent research. These applications have been developed to support various medical systems and other fields. However, there is a need for a comprehensive evaluation framework to determine the optimal SSVEP-based BCI application for specific criteria. One of the main challenges in the literature reviewed is the diversity of SSVEP-based BCI applications, with no clear consensus on the optimal approach. Studies have shown a range of variations in the developed applications, taking into account different criteria and ignoring others. It is crucial to consider the following criteria: security [28, 58, 64], robot control [52, 65–67], BCI framework [37, 56, 59], drone control [55, 66], and BCI speller [62] to design and implement an optimal SSVEP-based BCI application. The taxonomy presented in this review highlights that these criteria are crucial in developing SSVEP-based BCI applications. However, it is important to note that these five essential criteria should be considered together, and none should be neglected when evaluating developed SSVEP-based BCI applications. Another issue is that the evaluation criteria are comparative and may overlap with other models when considering multiple evaluation criteria and the importance levels of each [91, 92]. This highlights the need for a comprehensive evaluation framework that considers multiple criteria and their relative importance to determine the optimal SSVEP-based BCI application. Generally, evaluation criteria can be categorized into benefit and cost [93]. Benefit criteria refer to values considered more valuable, whereas cost criteria are the opposite. From a BCI perspective, the five evaluation criteria discussed in this review are considered to be benefit criteria. Therefore, due to the issues discussed, the evaluation and benchmarking of SSVEP-based BCI applications is a complex multicriteria decision problem. MCDM is a methodology used to evaluate alternatives and make decisions based on multiple, often conflicting criteria. MCDM involves various processes, including structuring, planning, and solving decision problems using multiple criteria [94, 95]. Therefore, MCDM methods could be useful for evaluating and selecting the optimal SSVEP-based BCI application based on the abovementioned criteria.

In summary, the main challenges of developing SSVEP-based BCI applications include ensuring security, achieving accurate robot control, designing an efficient BCI framework, ensuring precise drone control, and achieving high accuracy in BCI speller applications. These challenges are further complicated because different criteria may have varying levels of importance and overlap with other models. Therefore, developing a comprehensive evaluation framework using MCDM methods is necessary to address these challenges and benchmark the developed SSVEP-based BCI applications. This framework would involve decision-makers providing qualitative and/or quantitative assessments to determine the performance of each alternative concerning each criterion and the relative importance of the evaluation criteria concerning the overall objective [96–101]. Therefore, the propose solution for the above issues is explained in the next section.

## 8. Evaluation and Benchmarking of SSVEP-Based BCI Applications: Future Methodology

**Figure 10:**
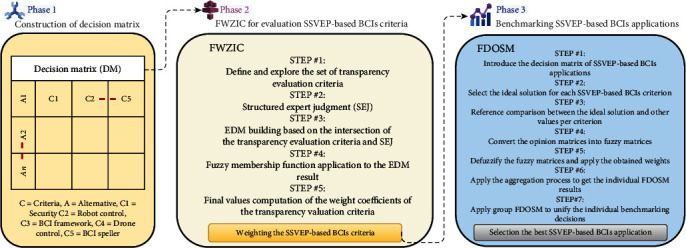
Proposed methodology for evaluation and benchmarking of SSVEP-based BCI applications for disabilities.

Fuzzy MCDM techniques intervene in clinical fields to provide intelligent decision-making [102–105]. Therefore, a new framework proposal can be developed as a trusted tool for future neurology and neuroscience research (i.e., disabilities). The methodology can be introduced in three phases, as shown in Figure 10.

### 8.1. Phase 1: Construction of Decision Matrix

This section explains the developed dynamic DM used to evaluate and benchmark SSVEP-based BCI applications. The developed applications must be benchmarked based on the five SSVEP-based BCI criteria: security, robot control, BCI framework, drone control, and BCI speller. Therefore, a new decision matrix must be developed for this purpose [106]. The decision matrix is the most critical aspect of the assessment and benchmarking technique [91, 92, 94, 107–109]. The primary decision-making components are the five affected SSVEP-based BCI criteria and alternatives (SSVEP-based BCI applications). The processes taken to construct the decision matrix are detailed in Table 1. In addition, two MCDM methods (FWZIC-FDSOM) must be formulated to evaluate and benchmark the developed SSVEP-based BCI applications using the developed decision matrix, as explained in the following sections.

### 8.2. Phase 2: FWZIC for Evaluation of SSVEP-Based BCI Criteria

**Figure 11:**
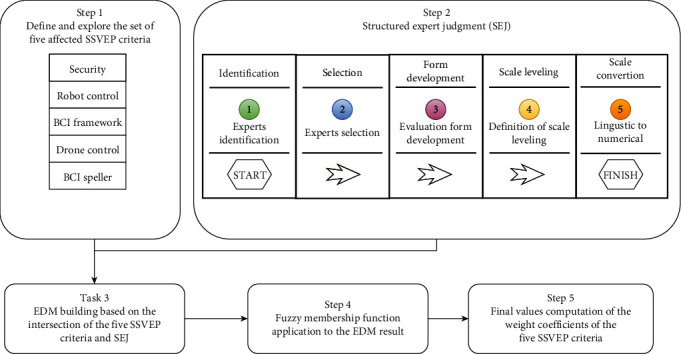
FWZIC methodology of evaluation of SSVEP-based BCI criteria.

FWZIC is one MCDM method that needs to be used for weighting the five SSVEP-based BCI criteria.  Figure 11 illustrates the steps of FWZIC through five essential processes that need to be applied for each set of five criteria [110, 111]. The five steps are illustrated below.

**Step 1 .**

Establish the set of evaluation of SSVEP-based BCI criteria: the predetermined set of assessment criteria of SSVEP-based BCIs should be examined and presented in the first step [92].

**Step 2 .**

Structured expert judgment (SEJ) is a process for identifying and selecting a team of experts in the SSVEP-based BCIs. The experts are nominated and selected, and a panel is formed [112]. The panel then uses a numerical scale to evaluate each of the five criteria, as shown in Table 2, using an evaluation form to capture the consensus of all panel members. In this process, a panel of four experts subjectively assesses the criteria.

**Step 3 .**

The expert decision matrix (EDM) is constructed based on the list of selected experts and their choices within specific criteria for SSVEP-based BCIs. The EDM consists of alternatives and decision criteria [113], as shown in Table 3. Each criterion in the attribute of SSVEP-based BCI criteria is cross-referenced with each expert (Ei), who evaluates the relevance of each criterion.

**Step 4 .**

**Figure 12:**
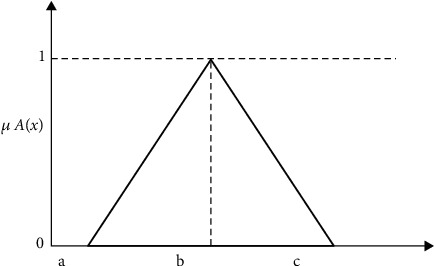
Membership of TFNs.

A fuzzy membership function and defuzzification procedure are applied to the data in the expert decision matrix (EDM) to improve the accuracy and usability of the data for criterion analysis in MCDM. In MCDM, assigning specific preference rates to each criterion is often difficult due to ambiguity and imprecision [114–116]. Using fuzzy techniques, such as triangular fuzzy numbers (TFNs), can address this issue by allowing for calculating relative values for criteria using fuzzy numbers rather than exact numbers. TFNs are a common type of fuzzy number and are represented as a, b, and c with a triangle membership function, as shown in Figure 12. They are preferred for their simplicity and are frequently used in real-world applications [117].

The membership function (*x*) of TFN *A* is given by

(1) $$\begin{array}{c} \mu \;A\left(x\right)=\left\{\begin{array}{c} \;0\text{ if }x<a,\\ \frac{x-a}{b-a}\text{ if }a\leq x\leq b,\\ \frac{c-x}{c-b}\text{ if }b\leq x\leq c,\\ \;0\text{ if }x>c, \end{array}\right. \end{array}$$

where *a* ≤ *b* ≤ *c*.

**Remark 1 .**

Let $\tilde{x}=\left(a1,b1,c1\right)$ and $\;\tilde{y}=\left(a2,b2,c2\right)$ be two nonnegative TFNs and *α* ∈ ℝ_+_. Following the extension principle, the arithmetic operations are defined as follows:

(2) $$\begin{array}{c} \;\tilde{x}+\tilde{y}=\left(a1+a2,b1+b2,c1+c2\right), \end{array}$$

(3) $$\begin{array}{c} \tilde{x}-\tilde{y}=\left(a1-c2,b1-b2,c1-a2\right), \end{array}$$

(4) $$\begin{array}{c} \alpha \tilde{x}=\left(\alpha a1,\alpha b1,\alpha c1\right), \end{array}$$

(5) $$\begin{array}{c} \tilde{x}-1≅\left(\frac{1}{c1},\frac{1}{b1},\frac{1}{a1}\right), \end{array}$$

(6) $$\begin{array}{c} \tilde{x}\times \tilde{y}≅\left(a1a2,b1b2,c1c2\right), \end{array}$$

(7) $$\begin{array}{c} \frac{\tilde{x}}{\tilde{y}}≅\left(\frac{a1}{c2},\frac{b1}{b2},\frac{c1}{a2}\right). \end{array}$$

The value of each numerical term with TFN is shown in Table 4.

Table 4 indicates that all linguistic variables should be transformed to TFNs, supposing that the fuzzy number is the variable for each expert N criterion. In other words, expert N should be asked to identify the critical degree of the assessment criteria (SSVEP-based BCIs) inside variables assessed using linguistic variables.

(1) By using Equation (8), the ratio of fuzzification data is determined. As demonstrated in Table 5, the preceding equations are employed with TFNs [117].
  (8) $$\begin{array}{c} \frac{\tilde{\text{Imp}\left(E1/C1\right)}}{\sum_{j=1}^{n}\tilde{\text{Imp}\left(E1/C_{1j}\right)}}, \end{array}$$
  where $\tilde{\text{Imp}\left(E1/C1\right)}$ represent the fuzzy number of Imp(*E*1/*C*1)
(2) To determine the final fuzzy values of the weight coefficients of the criterion ${\left(\tilde{w1},\tilde{w2},\cdots ,\tilde{wn}\right)}^{T}$, the mean values should be determined. The fuzzy EDM $\left(\tilde{\text{EDM}}\right)$ is utilized to calculate the final weight value of each SSVEP-based BCI criterion using Equation (9).
  (9) $$\begin{array}{c} \tilde{w_{j}}=\frac{\sum_{i=1}^{m}\left(\tilde{\text{Imp}\left(E_{ij}/C_{ij}\right)}/\sum_{j=1}^{n}\tilde{\text{Imp}\left(E_{ij}/C_{ij}\right)}\right)}{m},\text{for }i=1,2,3,..m\text{ and }j=1,2,3,..n. \end{array}$$
(3) Defuzzification to find the final weight: the centroid approach is the most prevalent defuzzification technique. Using TFNs, the mathematical expression for this procedure is (*a* + *b* + *c*)/3. Before computing the final values of the weight coefficients, the weight of importance should be allocated to each criterion based on the total weights of all SSVEP-based BCI criteria for the rescaling purpose used in this step

**Step 5 .**

Compute the final values of the weight coefficients of the evaluation criteria: in this stage, the final values of the weight coefficients for the evaluation criteria (*w*1, *w*2, ⋯,*w*5)^*T*^  that represented C1 = security, C2 = robot control, C3 = BCI framework, C4 = drone control, and C5 = BCI speller should be determined using the fuzzy data for the criterion from the previous step.

At this step, the weights for the five SSVEP-based BCI criteria will be calculated to be used with the FDOSM method in the next phase.

### 8.3. Phase 3: FDOSM Method for Benchmarking SSVEP-Based BCI Applications

**Figure 13:**
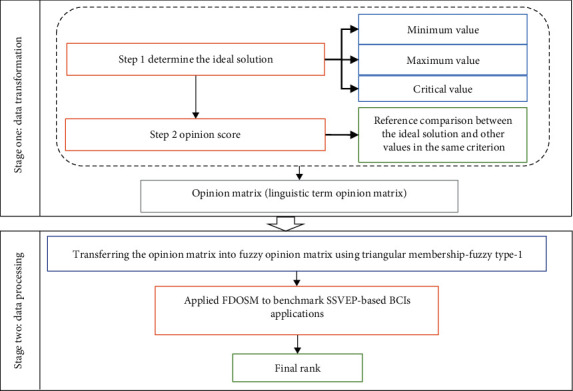
FDOSM stages [122].

The FDOSM is an MCDM method for ranking alternatives, such as in a benchmarking process for SSVEP-based BCI applications. FDOSM is a mathematical model that addresses MCDM issues involving individual and group decision-making contexts [29, 118, 119]. The FDOSM process consists of three units: a data input unit, a data transformation unit, and a data processing unit [120]. It also has two phases for group decision-making, external and internal aggregations [121], as illustrated in Figure 13. The steps of the FDOSM method can be summarized as follows:

- Data input unit: like existing MCDM approaches [122, 123], the proposed MCDM method assigns *m* choices to each MCDM issue. *A*_1_, ⋯, *A*_*m*_ presented SSVEP-based BCI applications and *n* set of decision criteria *C*_1_, *C*_2_ ⋯ , *C*_*n*_ represented SSVEP-based BCI criteria. The DM represents this block's output. Then, this choice matrix is converted into an opinion matrix
- Data transformation unit: once the DM has been constructed (as the output of the first block in the FDOSM process), the transformation unit is used to select a three-parameter optimal solution consisting of minimum, maximum, and critical values. The minimum value criterion is used for cost criteria, where the lowest value indicates the best option. The maximum value is used for benefit criteria, where the highest value represents the best solution. The critical value is used when the optimal solution is neither minimum nor maximum [124]. The following steps are then followed to complete this stage of the FDOSM process:

**Step 6 .**

Select the optimal solution (SSVEP-based BCI application). Consequently, the optimal solution should be described as follows:

(10) A∗=vijimaxj∈J,vijiminj∈J,Opij∈I.Ji=1.2.3⋯..m

**Step 7 .**

In the FDOSM process, the optimal solution is compared to the alternative values (remaining SSVEP-based BCI applications) based on the criterion, with weights implicitly assigned to the assessment criteria. Subjectively, the significance of the differences between the ideal solution and the alternatives can be evaluated, as shown in

(11) $$\begin{array}{c} \text{OPlang}=\left\{\left(\left({\tilde{v}}_{ij}⊗v_{ij}\right|j\in J\right).\left|i=1.2.3\right.\cdots ..\left.m\right)\right\}. \end{array}$$

A panel of three experts in data mining with the SSVEP-based BCI should be asked in this stage for more than five years of experience in this field.

#### 8.3.1. Data Processing Unit

The output of the transformation unit is referred to as the opinion matrix [109]. The final block of the FDOSM process involves converting the opinion matrix into a fuzzy opinion decision matrix using triangular fuzzy numbers (TFNs). A direct aggregation operator, such as the arithmetic mean, is then applied.  Table 6 shows the transformation of linguistic terms into TFNs after comparing the ideal solution with other values in the decision matrix.

**Step 8 .**

Once the opinion matrix is created in the FDOSM process, a fuzzification process is used to convert it to the fuzzy opinion decision matrix using triangular fuzzy numbers (TFNs). This is done by replacing the opinion terms with TFNs, which can be defined by their membership function as follows:

(12) $$\begin{array}{c} \mu A\left(x\right)=\left\{\begin{array}{c} 0\text{ if }x<a,\\ \frac{x-a}{b-a}\text{if }a\leq x\leq b,\\ \frac{c-x}{c-b}\text{if }b\leq x\leq c,\\ 0\text{ if }x>c, \end{array}\right. \end{array}$$

where *a* ≤ *b* ≤ *c*.

**Remark 2 .**

$\tilde{x}=\left(a1,b1,c1\right)$ and $\tilde{y}=\left(a2,b2,c2\right)$ are two nonnegative TFNs, and *α* ∈ ℝ_+_. The arithmetic operations are defined according to the extension principle as follows:

1. $\tilde{x}+\tilde{y}=\left(a1+a2,b1+b2,c1+c2\right)$
2. $\tilde{x}-\tilde{y}=\left(a1-c2,b1-b2,c1-a2\right)$
3. $\alpha \tilde{x}=\left(a\;a1,\alpha \;b1,\alpha \;c1\right)$
4. ${\tilde{x}}^{-1}≅\left(1/c1,1/b1,1/a1\right)$
5. $\tilde{x}\times \tilde{y}≅\left(a_{1}a_{2},b_{1}b_{2},c_{1}c_{2}\right)$
6. $\tilde{x}/\tilde{y}≅\left(a1/c2,b1/b2,c1/a2\right)$

**Step 9 .**

The fuzzy opinion decision matrix is subject to direct aggregation using an aggregation operator, such as the arithmetic mean, as described in [125]. The aggregation process can be completed using

(13) $$\begin{array}{c} A_{m\left(x\right)=\left(\sum \left(a_{f}+a_{m+}a_{1}\right)\left(b_{f}+b_{m}+b_{1}\right)\left(c_{\text{f}}+c_{m}+c_{1}\right)\right)/n}. \end{array}$$

**Step 10 .**

The centroid method can be utilized for the defuzzification process by applying the following equation:

(14) $$\begin{array}{c} \text{Diff}=\frac{\left(a+b+c\right)}{3}. \end{array}$$

The best-ranking order correlates to the lowest mean score value.

#### 8.3.2. External Aggregation

In external aggregation, fuzzy opinion matrices from various DMs are individually processed based on the processes outlined in the processing unit. The outcomes of the decision matrices should then be aggregated into the final group decision using the arithmetic mean. In this instance, the expert opinions will be jointed after determining the final ranking.

## 9. Conclusion

SSVEP-based BCI applications allow users to be in touch with the real world through repeated visual stimulation. The presentation of this study is oriented towards understanding the academic literature from various contextual aspects by considering the research domain from the technical and scientific parts. In this organized review, the study has stressed important facts about commonly used deep learning techniques for data processing in SSVEP-based BCIs such as RBM, RNN, LSTM, DNN, and CNN. Based on the analysis done, the research portrays that the CNN method has been applied more often in various domains, although the others provide higher accuracy levels. The advanced data acquisition, recording, and pattern recognition methods for deep learning in SSVEP-based BCI applications are discussed. Improvements to the deep learning technique can enrich the SSVEP-based BCI applications in many domains, streamline signal processing, allow additional targets, check loss of attention, and make allowances for independent BCI operation. Several requirements can help to ensure that deep learning technology in BCI applications based on SSVEP is trustworthy. These include thorough testing and evaluation, transparent design and decision-making processes, robust security and privacy measures, and ethical considerations. These requirements help to ensure that these systems are accurate, reliable, effective, and easy to use. By meeting these requirements, we can help to ensure that deep learning technology in these applications is safe, reliable, and trustworthy. The study outcome dramatically impacts the community of disabled people since it addresses the existing recommended solution to solve them in the future in a well-designed manner. Overall, deep learning is considered an approach that can be deployed in SSVEP-based BCI applications, which can aid disabled people. Other researchers can benefit from this study as it provides a clear guideline for selecting the best techniques for SSVEP-based BCI supported by scientific justification. Five SSVEP-based BCI criteria have been defined in this study that affects the development of SSVEP-based BCI applications: C1 = security, C2 = robot control, C3 = BCI framework, C4 = drone Control, and C5 = BCI speller. Accordingly, the selection process for the best SSVEP-based BCI applications cannot be achieved based on a specific criterion. In order to make the robust selection, researchers must be satisfied that the SSVEP-based BCI application will perform well on real-world data based on the relevant criteria (five predefined SSVEP-based BCI criteria). SSVEP-based BCI applications are validated by applying accuracy measures to a validation dataset. However, real-world data is frequently vastly different, and the evaluation metric may not reflect the product's intended purpose. In addition to such measurements, inspecting selection and their justifications is beneficial. Therefore, the five predefined SSVEP-based BCI criteria should be considered simultaneously for choosing the optimal SSVEP-based BCI application within other BCI fields. In these contexts, the presented study developed a proposed solution to overcome these issues and can be used as a trust framework for selecting SSVEP-based BCI applications for future neurology and neuroscience research. The future study of using deep learning technology in SSVEP-based BCI applications can focus on a number of areas. Some of the possible research directions include the following:

- Optimization and improvement of the current deep learning models for SSVEP recognition: this could involve the exploration of different deep learning algorithms and architectures to achieve higher recognition accuracy and lower computational cost
- Hybrid techniques: combining deep learning with other signal processing techniques (such as filtering, feature extraction, and classification methods) to improve the overall performance of the BCI system
- Real-time implementation: research could focus on developing deep learning-based BCI systems that can operate in real time without significant delay or latency
- Generalization: improving the ability of the deep learning models to generalize to new subjects and new SSVEP frequencies without the need for individualized model training
- Integration with other modalities: research could focus on integrating deep learning with other modalities, such as EEG and fMRI, on enhancing the information obtained from the BCI system and improve its accuracy
- Robotic rehabilitation applications: developing deep learning-based BCI systems for use in robotic rehabilitation systems to improve the quality of life for individuals with disabilities or injuries

These are just a few examples of the potential research directions in this field. The goal of future studies will be to further advance the state of the art in deep learning-based SSVEP-BCI applications and to make these systems more practical, efficient, and widely accessible.

## Figures and Tables

**Table 1:** The developed decision matrix.

Alternatives/criteria		SSVEP-based BCI criteria				
SSVEP-based BCI applications		C1	C2	C3	C4	C5
A1	Application #1	C1-A1	C2-A1	C3-A1	C4-A1	C5-A1
A2	Application #2	C1-A2	C2-A2	C3-A2	C4-A2	C5-A2
A3	Application #3	C1-A3	C2-A3	C3-A3	C4-A3	C5-A3
A4	Application #4	C1-A4	C2-A4	C3-A4	C4-A4	C5-A4
A5	Application #5	C1-A5	C2-A5	C3-A5	C4-A5	C5-A5
⋮	⋮	⋮	⋮	⋮	⋮	⋮
A*n*	Application #*n*	C1-A*n*	C2-A*n*	C3-A*n*	C4-A*n*	C5-A*n*

C = criteria; A = alternative; C1 = security; C2 = robot control; C3 = BCI framework; C4 = drone control; C5 = BCI speller.

**Table 2:** Five-point Likert scale and equivalent numerical scale.

Linguistic terms	Numerical scoring scale
Not important	1
Slight important	2
Moderately important	3
Important	4
Very important	5

**Table 3:** EDM.

Criteria/experts	*C*1	*C*2	*…*	*Cn*
*E*1	Imp (*E*1/*C*1)	Imp (*E*1/*C*2)	*…*	Imp (*E*1/*Cn*)
*E*2	Imp (*E*2/*C*1)	Imp (*E*2/*C*2)	*…*	Imp (*E*2/*Cn*)
*E*3	Imp (*E*3/*C*1)	Imp (*E*3/*C*2)	*…*	Imp (*E*3/*Cn*)
*...*	*…*	*…*	*…*	*…*
*Em*	Imp (*En*/*C*1)	Imp (*En*/*C*2)	*…*	Imp (*Em*/*Cn*)

Imp represents the importance level.

**Table 4:** Numerical terms and their equivalent TFNs.

Numerical scoring scale	TFNs
1	(0.00, 0.10, 0.30)
2	(0.10, 0.30, 0.50)
3	(0.30, 0.50, 0.75)
4	(0.50, 0.75, 0.90)
5	(0.75, 0.90, 1.00)

**Table 5:** Fuzzy EDM ($\tilde{\text{EDM}}$) [117].

Experts	Criteria			
	$\tilde{C1}$	$\tilde{C2}$	…	$\tilde{Cn}$
*E*1	$\frac{\tilde{\text{Imp}\left(E1/C1\right)}}{\sum_{j=1}^{n}\tilde{\text{Imp}\left(E1/C_{1j}\right)}}$	$\frac{\tilde{\text{Imp}\left(E1/C2\right)}}{\sum_{j=1}^{n}\tilde{\text{Imp}\left(E1/C_{1j}\right)}}$	…	$\frac{\tilde{\text{Imp}\left(E1/Cn\right)}}{\sum_{j=1}^{n}\tilde{\text{Imp}\left(E1/C_{1j}\right)}}$
*E*2	$\frac{\tilde{\text{Imp}\left(E2/C1\right)}}{\sum_{j=1}^{n}\tilde{\text{Imp}\left(E2/C_{2j}\right)}}$	$\frac{\tilde{\text{Imp}\left(E2/C2\right)}}{\sum_{j=1}^{n}\text{I}\text{mp}\left(E2/C_{2j}\right)}$	…	$\frac{\tilde{\text{Imp}\left(E2/Cn\right)}}{\sum_{j=1}^{n}\text{I}\text{mp}\left(E2/C_{2j}\right)}$
*E*3	$\frac{\tilde{\text{Imp}\left(E3/C1\right)}}{\sum_{j=1}^{n}\tilde{\text{Imp}\left(E3/C_{3j}\right)}}$	$\frac{\tilde{\text{Imp}\left(E3/C2\right)}}{\sum_{j=1}^{n}\tilde{\text{Imp}\left(E3/C_{3j}\right)}}$	…	$\frac{\tilde{\text{Imp}\left(E3/Cn\right)}}{\sum_{j=1}^{n}\tilde{\text{Imp}\left(E3/C_{3j}\right)}}$
*E*4	$\frac{\tilde{\text{Imp}\left(E4/C1\right)}}{\sum_{j=1}^{n}\tilde{\text{Imp}\left(E4/C4_{j}\right)}}$	$\frac{\tilde{\text{Imp}\left(Em/C2\right)}}{\sum_{j=1}^{n}\tilde{\text{Imp}\left(Em/C_{4j}\right)}}$	…	$\frac{\tilde{\text{Imp}\left(E4/Cn\right)}}{\sum_{j=1}^{n}\tilde{\text{Imp}\left(E4/C_{nj}\right)}}$

**Table 6:** Linguistic terms and their equivalent TFNs.

Linguistic terms	TFNs
No difference	(0.00, 0.10, 0.30)
Slight difference	(0.10, 0.30, 0.50)
Difference	(0.30, 0.50, 0.75)
Big difference	(0.50, 0.75, 0.90)
Huge difference	(0.75, 0.90, 1.00)
